# Deep Learning Approaches for the Classification of Keloid Images in the Context of Malignant and Benign Skin Disorders

**DOI:** 10.3390/diagnostics15060710

**Published:** 2025-03-12

**Authors:** Olusegun Ekundayo Adebayo, Brice Chatelain, Dumitru Trucu, Raluca Eftimie

**Affiliations:** 1 Laboratoire de Mathématiques de Besançon, Université Marie et Louis Pasteur, F-25000 Besançon, France; olusegun.adebayo@univ-fcomte.fr; 2 Service de Chirurgie Maxillo-Faciale, Stomatologie et Odontologie Hospitalière, CHU Besançon, F-25000 Besançon, France; bchatelain@chu-besancon.fr; 3 Division of Mathematics, University of Dundee, Dundee DD1 4HN, UK

**Keywords:** deep learning, classification, benign skin disorders, malignant skin disorders, keloid, dermatoscopic images, non-dermatoscopic images, transfer learning, CNN

## Abstract

**Background/Objectives:** Misdiagnosing skin disorders leads to the administration of wrong treatments, sometimes with life-impacting consequences. Deep learning algorithms are becoming more and more used for diagnosis. While many skin cancer/lesion image classification studies focus on datasets containing dermatoscopic images and do not include keloid images, in this study, we focus on diagnosing keloid disorders amongst other skin lesions and combine two publicly available datasets containing non-dermatoscopic images: one dataset with keloid images and one with images of other various benign and malignant skin lesions (melanoma, basal cell carcinoma, squamous cell carcinoma, actinic keratosis, seborrheic keratosis, and nevus). **Methods:** Different Convolution Neural Network (CNN) models are used to classify these disorders as either malignant or benign, to differentiate keloids amongst different benign skin disorders, and furthermore to differentiate keloids among other similar-looking malignant lesions. To this end, we use the transfer learning technique applied to nine different base models: the VGG16, MobileNet, InceptionV3, DenseNet121, EfficientNetB0, Xception, InceptionRNV2, EfficientNetV2L, and NASNetLarge. We explore and compare the results of these models using performance metrics such as accuracy, precision, recall, F1_*score*_, and AUC-ROC. **Results:** We show that the VGG16 model (after fine-tuning) performs the best in classifying keloid images among other benign and malignant skin lesion images, with the following keloid class performance: an accuracy of 0.985, precision of 1.0, recall of 0.857, F1 score of 0.922 and AUC-ROC value of 0.996. VGG16 also has the best overall average performance (over all classes) in terms of the AUC-ROC and the other performance metrics. Using this model, we further attempt to predict the identification of three new non-dermatoscopic anonymised clinical images, classifying them as either malignant, benign, or keloid, and in the process, we identify some issues related to the collection and processing of such images. Finally, we also show that the DenseNet121 model has the best performance when differentiating keloids from other malignant disorders that have similar clinical presentations. **Conclusions:** The study emphasised the potential use of deep learning algorithms (and their drawbacks), to identify and classify benign skin disorders such as keloids, which are not usually investigated via these approaches (as opposed to cancers), mainly due to lack of available data.

## 1. Introduction

Correctly identifying and classifying skin disorders is an important topic in the context of malignant vs. benign disorders, when patient treatment needs to start very quickly. There are various clinical examples where patients with certain malignant skin disorders have been misdiagnosed as benign disorders (because of their benign appearance), and thus they were not offered the appropriate treatment [1,2,3,4,5,6,7,8,9]. Such misdiagnoses are because clinical images of certain benign disorders are very similar to malignant cancers, and medical practitioners might not have the appropriate expertise to distinguish them.

In this study, we focus on identifying keloid lesions either as a benign skin disorder amongst different malignant and benign skin disorders, simply identifying keloids as keloids, or identifying keloids among other malignant lesions that look similar. Keloids are a particular type of fibroproliferative skin disorder characterised by increased deposition of collagen in the area of an initial wound (i.e., a raised “scar”) and the subsequent invasion of this lesion into the adjacent healthy tissue. This invasion aspect made researchers compare keloids with cancers [10,11,12,13]. Moreover, due to these differences and similarities between keloids and skin cancers [14], various studies and clinical case reports have mentioned the misdiagnosis of some skin cancers as keloids due to their similar clinical presentation; see, for example [1,4,5,7,9,15,16]. Also, the misdiagnosis of keloids as tumours can lead to improper surgical treatment that could lead to worse outcomes (i.e., patient disfigurement) [17]. Even if we acknowledge that such misdiagnoses are rare, there is the question (and opportunity) of using new computational approaches to improve the diagnosis of different benign and malignant skin disorders (when histopathology tests are not immediately available or when expert dermatologists are not immediately available).

We need to emphasise that the classical approach to diagnose various skin disorders uses dermatoscopy [18,19,20]. However, this medical device is perceived to be too complex and quite expensive to use [21,22]. Much less expensive options are classical point-and-shoot cameras or smartphone cameras that produce non-dermatoscopic images. However, the accuracy of the diagnostic results for various skin disorders (in particular for non-dermatoscopic images) is still not consistently optimal, and further research is needed to enhance its reliability and effectiveness in clinical practice [23]. With the rise of artificial intelligence, machine learning and deep learning approaches have been developed and employed to help clinicians diagnose different skin disorders and to provide faster and more accurate diagnostic results.

### Previous Work

Studies have shown that deep learning approaches perform better than machine learning approaches when it comes to image classification [24,25,26]. Thus, several studies have used deep learning approaches for biomedical image classification, e.g., lesion classification [27,28], breast cancer classification [29,30], etc., and for classifying benign and malignant skin disorders [31]. However, to the best of our knowledge, there are no studies that include the classification of keloids as benign or malignant skin disorders, nor do any studies exist involving their identification among benign and malignant skin lesions using non-dermatoscopic (clinical) image datasets.

Nevertheless, we note that there are studies that investigate keloids (and other scars) using machine learning and deep learning approaches. In [32], post-thyroidectomy scars (including keloid) were classified based on their severity. More precisely, the authors in [32] focused on images of post-thyroidectomy scars (which included hypertrophic scars and keloids), along with clinical features such as age, body mass index (BMI), and scar symptoms, and they used a convolutional block attention module integrated with a ResNet-50 model for the image-based severity prediction, achieving an accuracy of 0.733, AUC-ROC of 0.912, and a recall of 0.733 on an external test set when the images were combined with clinical data. However, more datasets are needed to improve model generalisation, and clinical validation is needed to ascertain the performance of the model. In [33], a cascaded vision transformer architecture was proposed for keloid segmentation and identification by focusing on the blood perfusion rate and growth rates of keloid lesions. The data used in this study were intensity and blood perfusion images of 150 untreated keloid patients, with images showing keloids from various body regions like the chest, face, and limbs obtained via Laser Speckle Contrast Imaging (LSCI), and they achieved an average accuracy of 0.927. However, more datasets are needed to improve model generalisation, and clinical validation is needed to ascertain the result of the model.

In addition to these very few keloid-focused studies, there are many more studies that focus on general tissue lesions (see also Table 1). For example, in [28] a novel Generative Adversarial Networks (GANs) architecture for generating medical images was introduced and used to generate synthetic liver lesion images (as opposed to the traditional data augmentation technique). They showed that adding these synthetically generated images to the original images (containing computed tomography images of 182 liver lesions) and training the model on these images improved the performance of their convolution neural network (CNN) model (approx. 7% increase in performance) compared to using the classical augmentation method available in deep learning, achieving an AUC-ROC of 0.93, a recall of 0.857, and a specificity of 0.924. A limitation to this study is that GAN-generated images may not fully capture real variations. Another is that the size of the dataset may prevent generalisation. In [27], a new non-dermatoscopic skin lesion dataset [34] (that has also been used in our current study) was introduced. The dataset included clinical images and related patient clinical information. The study in [27] showed that combining these patients’ clinical features (i.e., information) of non-dermatoscopic medical data with their images improved the performance of CNN models. They achieved an accuracy of 0.788±0.025, a precision of 0.8±0.028, and a recall of 0.788±0.025. Despite the promising result of the proposed model, the model may be too dependent on patient information, and the size of the data will likely cause the model not to generalise well, especially when tested on skin lesions found in anatomical regions not present or under-represented in the training dataset. In [31], a novel CNN model named SkinNet-16 was proposed for accurate early detection of skin lesions using two public dermatoscopic skin lesion images from [35,36]. The images were cleaned, feature extraction was done, and the proposed model was trained with the extracted features. The model achieved an accuracy of 0.992 and a recall of 0.98. The limitation of this study includes the fact that only a binary model was proposed despite the training dataset containing more that two skin lesions. Also, more training data might be needed for improved generalisation ability of the model.

In this study, we focus on CNN models, since they are the most widely used models. We acknowledge that there are other models such as vision transformers (ViTs) [37], which were originally used in natural language processing (NLP) and now are being used for image classification because they require fewer computations to achieve close enough performance compared to CNN models. Nevertheless, current studies show that they do not outperform CNN models except when trained on very large datasets [38,39]. For this reason (and because we do not have such large datasets), in this study we decided to focus exclusively on CNN models. The goal of our current article is threefold:

i. To train various CNN models to classify keloid images as benign lesions and not as malignant lesions (and for this, we use a large variety of skin lesion images, from keloids to melanoma, basal cell carcinoma, squamous cell carcinoma, seborrheic keratosis, nevus, etc., which could be either malignant or benign);
ii. To train the CNN models to classify images of various skin disorders in three separate classes: malignant, benign, or keloid;
iii. To train the CNN models to identify keloid images among other malignant lesions that can mimic keloids, e.g., basal cell carcinoma [40,41] and squamous cell carcinoma [42].

Note also that our focus in this study is on the following:

- The identification and classification of keloid lesions.
- Using non-dermatoscopic (clinical) images.

We aim to train the CNN models such that they can be used in communities where dermatoscopes are either rare or unavailable (and where dermatologists might not be available).

The paper is structured as follows. In Section 2, we discuss the data used for the experiments, the deep learning approaches considered, the data preprocessing steps, the architecture of the models, and the performance metrics. In Section 3, we discuss the results of the classification tasks for the three goals mentioned above using the performance metrics from Section 2 while highlighting the best models. In Section 4, we discuss the general overview of this study and its limitations. In Section 5, we present the conclusion, and in Section 6, we conclude with a summary of the results while also comparing them with some results from the published literature.

## 2. Materials and Methods

### 2.1. Data

**Figure 1:**
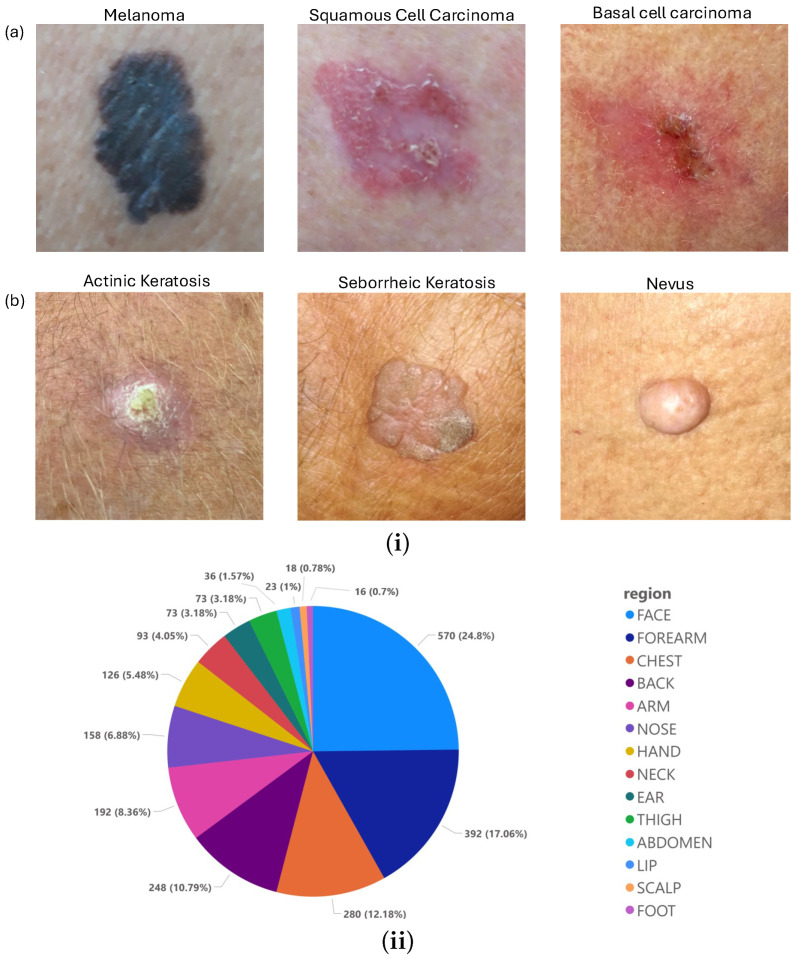
(**i**) A sample of the image dataset in [34] (the PAD dataset) for (**a**) malignant and (**b**) benign skin cancers. (**ii**) The anatomical regions where these skin lesions were found, as specified in [34].

**Figure 2:**
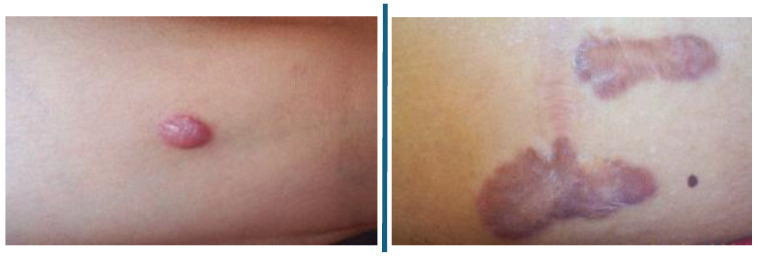
A sample of the cropped keloid images from the Kaggle dataset (see [44]). Note that this keloid dataset did not contain any information about anatomical regions where the keloid lesions were found.

In this study, we made use of two non-dermatoscopic datasets:

- The first dataset was taken from [34] and was obtained using a smartphone-based application created to help doctors and medical students collect clinical photos of skin lesions, as well as clinical information about the patient. Each dataset sample contains up to 26 attributes: a clinical diagnosis; a picture of the lesion; the patient’s age; the location of the lesion; if the lesion itches, bleeds, or has bled, hurts, has recently risen, has altered its pattern, is elevated, etc. The dataset contains 2298 images taken from 1373 patients between 2018 and 2019, which are available in Portable Network Graphics (PNGs) in raw format (i.e., just as they were taken). Each image in this dataset focused on one of the following six common skin disorders (also summarised in Table 2): melanoma, basal cell carcinoma, squamous cell carcinoma (which are considered malignant), actinic keratosis, seborrheic keratosis, and nevus (which are considered benign). We emphasise that 58.4% of the skin lesions and 100% of the skin cancers in this dataset are histopathology-confirmed (and this is about the same percentage as for the ISIC [43] dataset). For more information on the data, see [34].
  Figure 1i shows an example of some of the images from the first image dataset [34] divided into their respective pathological classification (i.e., either malignant or being), while Figure 1ii shows the anatomical region of these lesions.
- The second dataset is taken from [44] and consists of different non-dermatoscopic keloid images; we chose 274 keloid images and cropped them to ensure that these images are consistent with the (zoomed-in) images in the first dataset. Figure 2 shows a sample of the (keloid) images in this second dataset [44].

Note that there are studies that have shown that some of the skin disorders classified as benign in Table 2 may become malignant [45,46]; e.g., in [45], the authors showed that seborrheic keratosis can transform into squamous cell carcinoma, and in [46], the authors reported cases of malignant dermatofibroma. In this study, we categorised each of these skin disorders as either malignant or benign, as shown in Table 2. Furthermore, we categorised the keloids as “benign“ for the first classification task and as “keloids” for the second classification task.

### 2.2. Deep Learning Approaches

While the classical dense (i.e., fully connected) layers were the first used in deep learning, they have been largely replaced by convolutional neural networks (CNNs, also known as covnets), as they have been shown to perform better than densely connected layers [24]. One of the reasons for which convolutional layers tend to perform better is because they can learn local patterns from their input feature space compared to dense layers that learn global patterns. Also, the patterns learned by a convolutional layer are translation-invariant, while those of dense layers are not [24].

Due to the high computational cost of training new algorithms with high predictive performance, we decided to use transfer learning approaches for nine different already-published deep learning models—VGG16 [47], InceptionV3 [48], DensNet121 [49], MobileNet [50], EfficientNetB0 [51], Xception [52], InceptionRNV2 [53], EfficientNetV2-L [54], and NASNet-L [55]—which have been shown to perform well on biomedical image classification [56,57,58,59,60,61,62,63,64]. These models were pretrained on the Imagenet database, and in this study, we used them as base models. In the beginning, the weights of each base model were frozen, and the models were only trained on an additional dense input layer with 1024 neurons after a 2D global average pooling was done, followed by a unit dense output layer with a sigmoid activation function, as appropriate for binary classification. For a detailed description of these models, see Section 2.2.2 below.

#### 2.2.1. Data Preprocessing

In the first step, we loaded images available in the first dataset [34], which sums up to a total of 2298 coloured images with pixel values ranging between 0 and 255. We then recategorised the datasets based on the “diagnostic“ feature in relation to each image identified with their “im_id” feature. Next, we split the dataset into train, validation, and test sub-sets using stratified shuffle split from scikit learn library to preserve the percentage of the classes as in the original dataset (i.e., this makes sure the split data contain the proportionate percentage of the skin disorders when compared to the original dataset). We also applied the same split method to the second dataset (containing only keloid lesions) [44] and then added them together. The dataset was then split into train (80%) and test (20%) sets, while 90% of the train set was used for training, and 10% was used to validate the model. On the whole dataset, we applied the following standard preprocessing rules: (i) we rescaled each pixel value for each image matrix to lie between 0 and 1; (ii) we resized each image to a target size of our choice (i.e., 128×128). We then categorised each skin disorder as malignant or benign: melanoma, basal cell carcinoma and squamous cell carcinoma were classified as malignant [65] (a total of 1089 such images), while seborrheic keratosis, nevus, and actinic keratosis were classified as benign [66] (a total of 1209 such images).

Since the dataset was imbalanced and small, we first applied random oversampling on the train set to tackle the class imbalance issue and increase the dataset. We also applied data augmentation techniques, e.g., image flip, rotation, zoom, shift, and shear, to a random sample of each class in the original train dataset to introduce some variability and also increase the size. It is important to note that the oversampling and data augmentation methods detailed above were only applied to the train sets, while we kept the original images in the validation and test sets.

#### 2.2.2. Model Details

**Figure 3:**
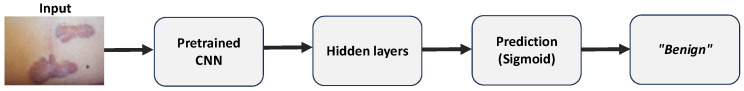
The transfer learning framework.

Building an efficient custom image classification CNN model from scratch with high accuracy is computationally demanding. Also, insufficient data might lead to inaccurate categorisation, and CNN training could take a while to converge [67]. Hence, most image classification tasks employ transfer learning methods, where already-trained models (considered base models) are reused on new datasets. Figure 3 shows the transfer learning framework used in this study: we removed the top layers containing the classification layers and replaced them with a hidden classification block (containing a dense layer(s) followed by an output layer depending on the number of classes).

**Figure 4:**
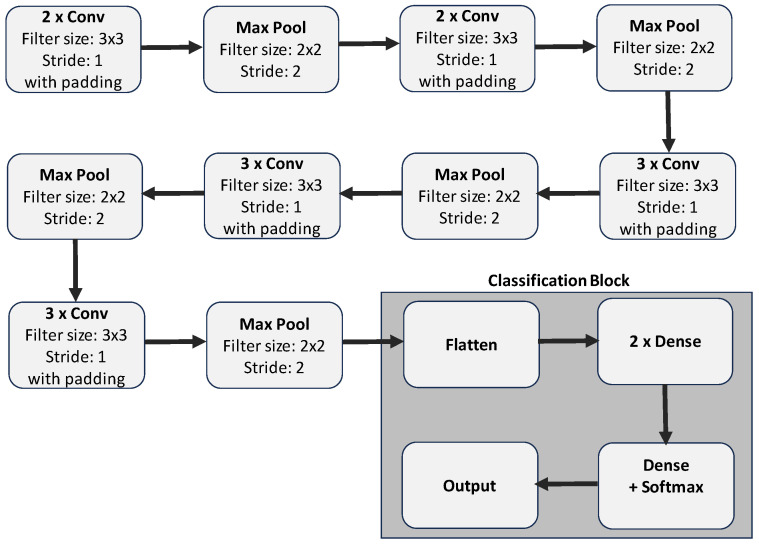
VGG16 architecture [49] highlighting the classification block, which is usually replaced during transfer learning.

**Figure 5:**
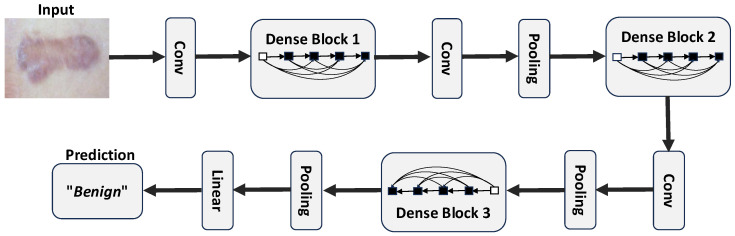
An example of a deep DenseNet architecture with three dense blocks [49], and with a keloid image as input. The layers between two adjacent blocks are referred to as transition layers.

**Figure 6:**
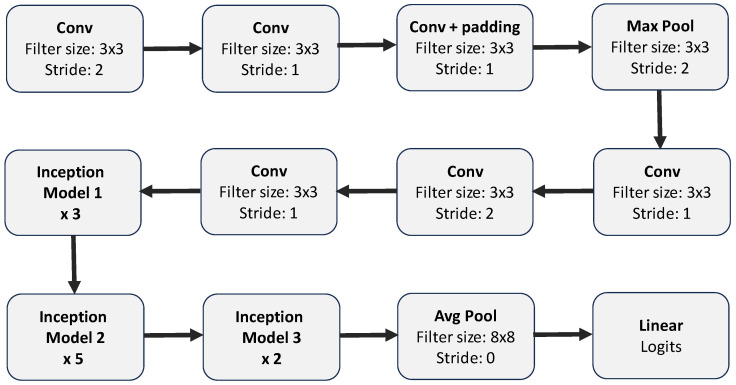
Summary of Inception-V3 model architecture (see also Table 5. For explicit details on the structure of Inception Model 1, Inception Model 2, and Inception Model 3, see [48].

Below, we introduce the models considered in this study:

1. **VGG16**: VGG16 was proposed in [47] by the Visual Geometry Group (VGG) at the University of Oxford. It consists of 13 convolutional layers (with 3×3 filter sizes), 5 max-pooling layers, and 3 dense (i.e., fully connected) layers including the output layer. The rectified linear unit (ReLU) activation function is used for each convolutional and dense layer except for the output layer, which uses the “softmax” activation function. Each convolutional layer use a 3×3 convolutional filter with a stride of 1 and same padding, which makes the resulting feature maps retain the same spatial dimensions as the input. The convolutional layers are stacked on each other, with the number of input filters doubled after each max-pooling layer (with a stride of 2). The depth of the convolutional layers is also increased monotonically. Figure 4 and Table 3 show a summary of the VGG16 architecture.
2. **DenseNet121**: This model, which was proposed in [49], is a feedforward network that connects all layers to all other layers in a feedforward fashion. It comprises 121 layers and consists of densely connected convolutional layers within dense blocks, promoting feature reuse and gradient flow. The model also consists of transition layers which help to control the growth of complexity between blocks. Figure 5 and Table 4 show a summary of the DenseNet121 architecture.
3. **InceptionV3**: InceptionV3 is an extension of GoogleNet (a model that has been shown to demonstrate strong classification performance in some biological applications [68,69]). InceptionV3 uses the inception model to minimise the number of parameters required to be trained, thus lowering the computational cost by concatenating numerous convolutional filters of various sizes into a new filter. Figure 6 and Table 5 summarise the InceptionV3 architecture.
4. **MobileNet**: The MobileNet model uses depthwise separable convolutions (a form of factorised convolutions that factorise a classical convolution into a depthwise convolution and a 1×1 convolution called a pointwise convolution) to reduce computational cost and model size. In one step of these convolutions, the depthwise convolution applies a single filter per input channel, and the pointwise convolution applies a 1×1 convolution to combine the outputs of the depthwise convolution. For more details on MobileNet, see [50]. Table 6 summarises the MobileNet architecture.
5. **EfficientNetB0**: EfficientNet is a family of CNNs that proposed a new scaling method after carefully identifying that better accuracy could be achieved when the depth, width, and resolution of the network are carefully balanced. The proposed scaling approach uses a simple compound coefficient to uniformly scale the depth, width, and resolution to reduce the computational cost and the model size. EfficientNet consists of 8 models in the range *B*0–*B*7, where the *B*1–*B*7 models are scaled increasingly from the baseline B0 model using different compound coefficients. The baseline model EfficientNetB0 is based on mobile inverted bottleneck convolution (MBConv) blocks [70]. In this study, we focus only on the baseline model (EfficientNetB0) due to limited computational resources. For more details on EfficientNet, see [51]. Table 7 summarises the EfficientNetB0 architecture.
6. **Xception**: The Xception model uses depthwise separable convolutions like MobileNets and the Inception models described above. However, this model is based on the hypothesis that cross-channels correlations and spatial correlations mappings in the feature maps of CNNs can be decoupled entirely. This is a stronger assumption than that of the Inception models, and hence the name “Xception” that derived from the phrase “Extreme Inception”. For more details on the Xception model, see [52]. Table 8 summarises the Xception architecture.
7. **InceptionRNV2**: This is the second verion of the InceptionResNet model. It is largely similar to the Inception model already described above (see InceptionV3). However, in InceptionResNet (i.e., a residual version of the Inception model), a cheaper Inception block is used preceded by a filter expansion layer which scales up the dimension of filter bank, hence cushioning the effect of the the dimensionality reduction caused by the Inception block. Another distinction between the InceptionResNet models and the vanilla Inception models is that batch normalisation is applied only to the standard layers not the summations, hereby increasing the overall number of Inception blocks. In this study, we used the second version of the InceptionResNet model. For more details on the InceptionResNet model, see [53]. Table 9 summarises the InceptionResNetV2 architecture.
8. **EfficientNetV2-L**: This is an extension of the EfficientNet models described above (see 5) with faster training time, because it uses about half the number of parameters used in EfficientNet. In this new family of models, the scaling approach introduced in [51] was combined with a training-aware neural architecture search (NAS) to optimise the training speed and number of parameters. Unlike the original EfficientNet, which uses depthwise separable convolutions, the fused-MBConv block fuses the initial pointwise and depthwise convolutions, reducing the computational cost. As opposed to the vanilla EfficientNet, EfficienNetV2 utilises both MBConv and the fused-MBConv [71] in the early layers. This new family comprises three variants (i.e., small, medium, and large) based on their size and performance. In this study, we made use of the largest variant (i.e., EfficientNetV2-L). For more details on EfficientNetV2, see [54]. Table 10 summarises the EfficientNetV2-L architecture.
9. **NASNet-L**: This model was designed using a proposed search space called NAS (that enables transferability) to find optimal network architectures. It utilises reinforcement learning to explore a preset search space of architectures while optimising performance and efficiency and also using a regularisation technique called “ScheduledDropPath”. It has different variants, NASNet-A, B, and C, tailored for different use cases (where A is the most accurate and was designed to deliver high performance, while B and C provide a trade-off between efficiency and accuracy, with B being more accurate than C). The model also includes large and small versions depending on the resources available. The large model (i.e., NASNet-L) is particularly effective for high-performance, while the small model (i.e., NASNetMobile) is particularly effective for resource-constrained environments like mobile devices. In this study, we used NASNet-L. For more details on NASNet models, see [55]. Table 11 summarises the NASNet-L architecture.

Generally in transfer learning, we replace the classical output layer of each pretrained model (i.e., 1000 output units) with the number of target classes $n_{c}$ that our classification tasks require: usually $n_{c}-1$ for binary classification and $n_{c}$ for multiclass classification.

In this experiment, we used the pretrained models as feature extractors and built a new neural network on top of them for our specific classification tasks: one binary class classification (benign vs. malignant lesion) and one multiclass classification (here with three classes: benign vs. malignant vs. keloid). We began the process by first initialising a base model (i.e., VGG16, DenseNet121, InceptionV3, MobileNet, EfficientNetB0, Xception, InceptionRNV2, EfficientNetV2-L, or NASNet-L), with weights pretrained on the ImageNet dataset. As already explained above, we excluded the fully connected layers at the top of the network (with output unit 1000), and specified the input shape parameter, which defines the shape of the input data (which we chose to be 128×128×3 for our tasks, where “3” corresponds to the number of channels in the images, as given by the RGB format). It is important to note that images were in the RGBA format (i.e., 4 channels) and were then converted to the RGB format before use for uniformity in the input shape.

Subsequently, we froze the weights of all layers in the pretrained model (i.e., we did not train them on the new data) and added these base layers to a new output block. This new output block is a Sequential model to which the pretrained model is added. We incorporated the following into this new model: a GlobalAveragePooling2D layer (to reduce the spatial dimensions of the output from the pretrained model), a dense layer with 1024 units, a ReLU activation function (to introduce non-linearity and learn high-level features), and a dropout layer with a rate of 0.5 included to prevent overfitting (by randomly setting half of the input units to zero during training). Lastly, a dense output layer with 3 (or 1) unit(s) and a softmax (or sigmoid) activation function were added for the multiclass (or binary) classification task, where 3 corresponds to the number of target classes for the multiclass classification, and 1 corresponds to binary classification (i.e., two target classes). We made use of the binary cross-entropy loss function for the binary classification task and the categorical cross-entropy loss function for the multiclasss task. Throughout these experiments, we made use of the Adam optimiser with a learning rate equal to 0.0001 for model training without fine-tuning and a rate of 0.00001 for model training with fine-tuning. We trained all the CNN models over 50 epochs with a batch size of 32.

When training the models, we considered the following cases regarding the base models and the train dataset:

**Base model:**

1. Freezing the base model (i.e., training only the weights and biases of the added model).
2. Fine-tuning the base model by unfreezing all layers of the base model (i.e., training the weights and biases of the base model alongside the added model).

**Train dataset:**

1. Training the model on the original train data after splitting it into train, validation, and test datasets.
2. Training the model on an oversampled train dataset.
3. Training the model on an augmented train dataset.

Note that for each category of the train dataset considered above, we also considered the two cases as regarded the state of the base model above.

#### 2.2.3. Evaluation Metrics

For the classification of images with the different models, we employed the following evaluation metrics that are given in terms of true positive (TP) values, true negative (TN) values, false positive (FP) values, and false negative (FN) values: accuracy, precision, recall, ${F1}_{score}$, AUC-ROC and the confusion matrix. For more details on the these performance metrics, see Appendix A. Note that each model was evaluated only on the out-of-sample dataset, i.e., the test dataset, which had not been exposed to the model at all during the training procedure.

## 3. Results

Here, we discuss the results obtained after applying the transfer learning technique using the pretrained CNN models as base models. We start with a binary classification in Section 3.1, followed by multiclass classifications in Section 3.2 and Section 3.4. Regarding the results in the tables below, we note that the values obtained for some of the pretrained models improved as we fine-tuned them.

### 3.1. Binary Classification: Benign vs. Malignant Lesions

First, we present the performance of the CNN models trained on the original dataset (i.e., before oversampling or applying data augmentation) on the test dataset. Table 12 shows the performance of the CNN models before fine-tuning the base models, while Table 13 shows their performances after fine-tuning (i.e., training all layers of the base models alongside the output layers).

In Table 12, we observe that MobileNet outperformed the rest of the models in accuracy, recall, and ${F1}_{\mathrm{score}}$. This was followed by DenseNet121 on the same metrics, but this one yielded the highest AUC-ROC score. VGG16 had the highest precision and only performed worse than DenseNet121 in terms of AUC-ROC measure. EfficientNetB0 and EfficientNetV2L had zero precision, recall, and ${F1}_{\mathrm{scores}}$; also, their accuracy and AUC scores were low and close to each other, making them the worse models. The next-worse models were the NASNetLarge and the Inception models (i.e., InceptionV3 and InceptionRNV2). InceptionV3, however, performed better than VGGG16 in terms of recall rate. In Table 13, we see that after fine-tuning, VGG16 outperformed the rest of the models in accuracy, recall, ${F1}_{score}$, and AUC-ROC. DenseNet121 had the highest precision rate and the second-best in AUC-ROC score. We also see that after fine-tuning, the overall performance of MobileNet reduced for almost all metrics except in terms of precision, where it increased slightly. In general, the performance of the other models seems to have increased after this fine-tuning, with the exception of MobileNet. Also, we see that the precision, recall, and ${F1}_{scores}$ of EfficientNetB0 and EfficientNetV2L went from zero to over 77%, implying that their initial weights and biases (i.e., initialised from ImageNet) performed really poorly on the test dataset. Now, the EfficientNetV2L had the second best accuracy and recall score, while the NASNetLarge model had the highest recall score. The Xception model seems to have also performed well, ranking second best in terms of precision.

Next, we trained the CNN models on the oversampled data (where we randomly increased the number of images for the classes with a lower number of images by adding copies of these randomly selected images). In Table 14, we show their performance on the test dataset without fine-tuning (i.e., without training the layers of the base models), while in Table 15, we show their performance after fine-tuning (i.e., training all layers of the base models). We see from Table 14 that VGG16 had the highest accuracy, and MobileNet had the highest recall and ${F1}_{score}$ while sharing the same accuracy with DenseNet121. VGG16 had the highest AUC-ROC, followed by DenseNet121 and MobileNet. EfficientNetB0 had the lowest recall, ${F1}_{score}$, and AUC-ROC score, while EfficientNetV2L had the lowest accuracy and precision.

After fine-tuning (Table 15), we see that VGG16 outperformed the other models only in AUC-ROC. EfficientNetB0 had the highest accuracy and the third best AUC score, while NASNetLarge had again the highest recall score. Hence, overall, VGG16 seems to have the highest ability to distinguish between malignant and benign skin lesions while possessing a good trade-off between precision and recall (see the high ${F1}_{score}$).

Finally, we trained the CNN models on the augmented data (obtained by applying rotations, flips, zoom-ins, and shears to randomly selected images), and we present their performance on the test data. This data augmentation approach not only increases the number of images available for training (to solve the class imbalance issue) but also introduces variability in the train dataset. In Table 16, we present the performance of these models (trained on the augmented dataset) before fine-tuning. Here, we see that MobileNet outperformed the other models in all metrics except recall, and it possesses the best trade-off between precision and recall. In contrast, EfficientNetB0 had the worst performance on all metrics.

Finally, in Table 17 we show the performance these CNN models (trained on augmented data) after the fine-tuning of base models. We see that VGG16 outperformed the other models in accuracy, precision, and AUC-ROC followed by EfficientNetV2L, which yielded the same accuracy, the highest ${F1}_{score}$, and was the second best in terms of recall and AUC-ROC.

Overall, VGG16 showed the best performance on test data after fine-tuning when the model was trained on raw training data (i.e, Table 13), oversampled training data (i.e., Table 15), and augmented train data (i.e., Table 17).

**Figure 7:**
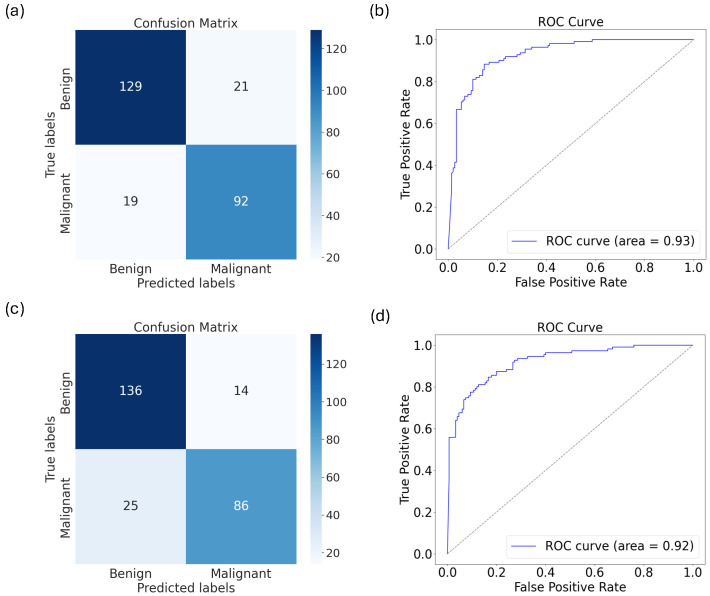
The (**a**) confusion matrix and (**b**) AUC-ROC curve for VGG16 model trained and validated on the original train and validation datasets, respectively (while fine-tuning the base model), and tested on the out-of-sample data (i.e., test data). (**c**) The confusion matrix and (**d**) AUC-ROC curve for VGG16 model trained on augmented training data (while fine-tuning the base model).

In Figure 7, we present the confusion matrices and AUC-ROC curves of the two best models: (a,b) show the VGG16 model trained on the original dataset and fine-tuned; (c,d) show the VGG16 model trained on the augmented dataset and fine-tuned.

### 3.2. Identifying Keloids as Keloids and Not Only as Benign vs. Malignant Skin Disorders

In what follows, we proceedd to train the models above not only to identify keloids as “benign“ lesions but also to be able to distinctively identify keloids as “keloids” among the various images of benign and malignant skin lesions. To this end, we trained the models again (using transfer learning while maintaining the architecture for multiclass classification, as described in Section 2.2.2) on the two datasets (i.e., the malignant vs. benign lesions, as classified in Table 2 and the keloid lesions, as shown in Figure 2), and fine-tuned them.

We present their performances when trained on the original training dataset, oversampled training dataset, and augmented training dataset:

(a) **Original training data**: In Table 18, we present the performance of the 10 models considered in this study (on test data) when they were trained for 50 epochs on the original training dataset validated on the original validation dataset (while fine-tuning all layers of the base models). We see here that VGG16 equally outperformed the rest of the model on all metrics, followed by Xception and DensNet121, respectively.
(b) **Oversampled training data**: In Table 19, we present the performance of the trained model on oversampled data. Here, we see that the performance of each of the models improved in comparison to Table 18. Here again, VGG16 performed better than the rest of the models on all metrics followed by Xception (with a slightly lower AUC that DenseNet121) and DenseNet121.
(c) **Augmented training data**: In an attempt to further improve the result of the models, instead of random oversampling, we applied data augmentation (such as rotations, flips, zooms, etc.) to increase the training dataset to introduce variability and to help the model generalise better. In Table 20, we see an increase in the performance of all the models as expected in comparison to Table 18 and Table 19. We see again that VGG16 outperformed the rest of the models on all metrics followed by EfficientNetV2L and InceptionV3, which both had lower AUC ROC scores compared to DenseNet121, which had the second best AUC ROC score, while its other performances ranked below InceptionV3.
  We emphasise here that out of the pretrained CNN models used, MobileNet model was the worst-performing model on the test data considered in this study.

**Table 18:** Accuracy, precision, recall, ${F1}_{score}$, and AUC of the models (rows 1–9) on the test dataset. Here, we trained and validated these models on the original train and validation datasets and *fine-tuned* the base models. In the “Accuracy” column, we show in bold the highest value indicating the best model.

Model	Accuracy	Precision	Recall	${F1}_{\mathrm{score}}$	AUC
VGG16	**0.8276**	0.8301	0.8276	0.8276	0.9376
MobileNet	0.7471	0.7534	0.7471	0.7480	0.8914
DenseNet121	0.7931	0.7942	0.7931	0.7922	0.9305
InceptionV3	0.7816	0.7841	0.7816	0.7823	0.9095
EfficientNetB0	0.7624	0.7663	0.7624	0.7632	0.8925
Xception	0.8199	0.8245	0.8199	0.8202	0.9309
InceptionRNV2	0.7854	0.7854	0.7854	0.7854	0.9091
EfficientNetV2L	0.7778	0.7843	0.7778	0.7743	0.9111
NASNetLarge	0.6743	0.7266	0.6743	0.6685	0.8665

**Table 19:** Accuracy, precision, recall, ${F1}_{score}$, and AUC of the models on the test dataset. Here, we trained these models on oversampled train dataset and validated them on the original validation datasets. The base models were *fine-tuned*. In the “Accuracy” column, we show in bold the largest value indicating the best model.

Model	Accuracy	Precision	Recall	${F1}_{\mathrm{score}}$	AUC
VGG16	**0.8506**	0.8530	0.8506	0.8502	0.9427
MobileNet	0.7625	0.7650	0.7625	0.7634	0.9032
DenseNet121	0.8084	0.8101	0.8084	0.8091	0.9295
InceptionV3	0.8008	0.8056	0.8008	0.8015	0.9205
EfficientNetB0	0.7969	0.7989	0.7969	0.7969	0.9148
Xception	0.8199	0.8239	0.8199	0.8205	0.9282
InceptionRNV2	0.8007	0.8036	0.8008	0.8008	0.9215
EfficientNetV2L	0.7969	0.7983	0.7969	0.7973	0.9179
NASNetLarge	0.7050	0.7222	0.7050	0.7061	0.8728

**Table 20:** Accuracy, precision, recall, ${F1}_{score}$, and AUC of the models (rows 1–9) on the test dataset. The models were trained on the augmented train dataset and validated on the original validation datasets, with the base models fine-tuned.

Model	Accuracy	Precision	Recall	${F1}_{\mathrm{score}}$	AUC
VGG16	**0.8774**	0.8813	0.8774	0.8778	0.9519
MobileNet	0.8046	0.8045	0.8046	0.8046	0.9240
DenseNet121	0.8391	0.8399	0.8391	0.8394	0.9452
InceptionV3	0.8467	0.8474	0.8467	0.8470	0.9403
EfficientNetB0	0.8429	0.8422	0.8429	0.8424	0.9406
Xception	0.8046	0.8072	0.8046	0.8046	0.9334
InceptionRNV2	0.8199	0.8222	0.8199	0.8206	0.9359
EfficientNetV2L	0.8467	0.8502	0.8467	0.8470	0.9322
NASNetLarge	0.8391	0.8410	0.8391	0.8394	0.9365

**Figure 8:**
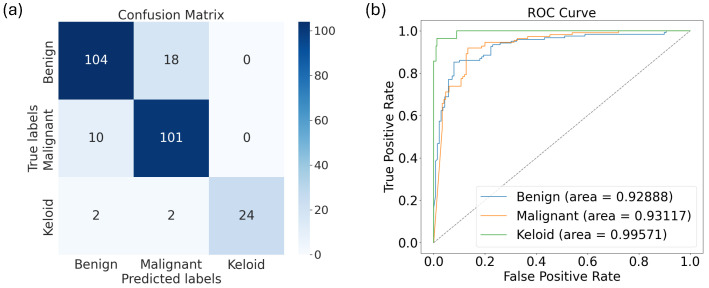
The (**a**) confusion matrix and (**b**) AUC-ROC curve for VGG16 trained on the augmented train data validated on the original validation dataset (while fine-tuning the base model) and tested on the test data.

Finally, we show in Figure 8 the confusion matrix and AUC-ROC curves for the best model: the VGG16 model trained on the augmented data.

From the confusion matrix and the AUC-ROC curves, we see that the model’s ability to distinguish keloids from the other classes is better than its ability to distinguish the other classes (i.e., the green curve in Figure 8b), though it also performed excellently well in distinguishing malignant and benign skin disorders individually, with its AUCs equal to 0.9294 and 0.9289, respectively.

### 3.3. New Test Data: Clinical Images

**Figure 9:**
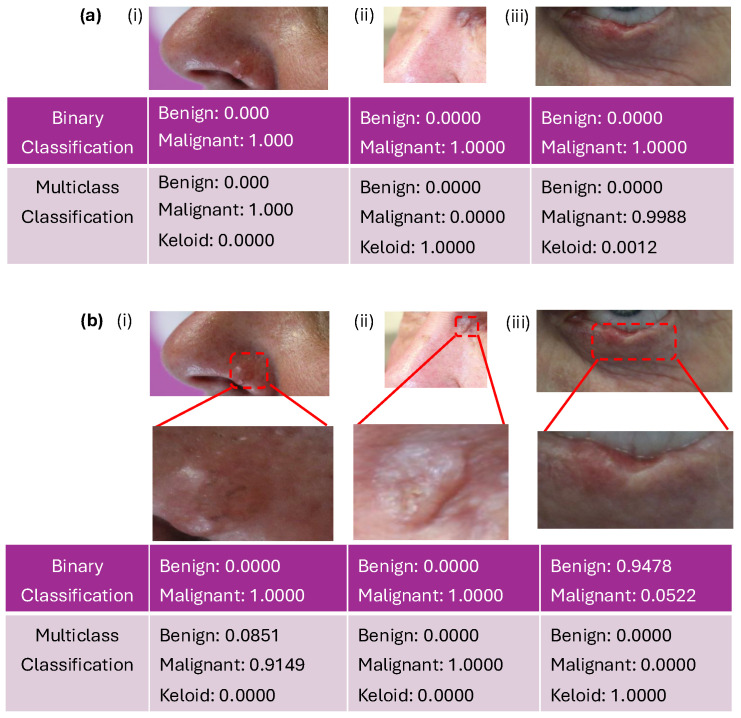
The result of our proposed binary model and multiclass model on (**a**) three anonymised clinical images of skin lesions (denoted by (**i**–**iii**)) and (**b**) their zoom-ins. The first rows of the tables in (**a**,**b**) show their class probabilities when tested with the proposed binary model, while the second rows of (**a**,**b**) show their class probabilities when tested with the proposed multiclass model.

To conclude the testing of our algorithm, we have finally used three new clinical anonymised images (see Figure 9a provided by B. Chatelain). To classify them, we used the our proposed algorithms (i.e., the VGG16 model trained to classify malignant vs. benign images, as well as the VGG16 model trained to classify malignant vs. benign vs. keloid images). To this end, we zoomed-in the original images (see Figure 9b) to be consistent with the other images in the datasets. We can see that the first two images, i.e., Figure 9b(i,ii), have been correctly identified in both cases as malignant. In contrast, the third zoomed-in image (i.e., Figure 9b(iii)) has been incorrectly identified as “benign“ in one case and as “keloid” in the second case.

Since trained clinicians can diagnose the lesions from a distance, we emphasise that we also tried to classify the original images (non-zoomed-in; see Figure 9a). In that case, we obtained a correct result with the binary algorithm for Figure 9a(i,iii). In contrast, with the multiclass algorithm, we obtained a correct prediction for Figure 9a(i,iii) but an incorrect result for Figure 9a(ii).

**Remark** **1.** *There were a few issues with the classification of the new images in Figure 9. First, the original images were taken at a distance, and to use the previous algorithms, we had to crop them so we could zoom-in to focus on the lesions (since the images we trained our algorithm on were focused; see Figure 1i). But this zoom-in led to blurred images.*

**Figure 10:**
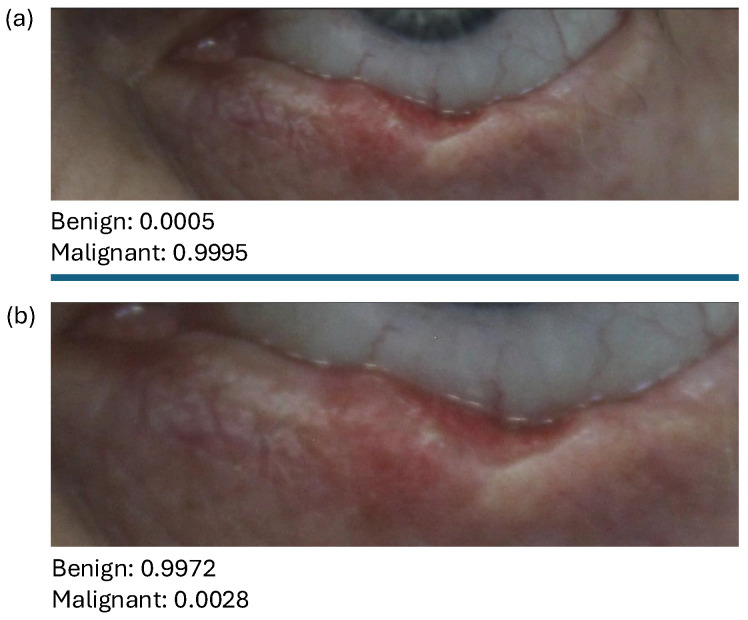
Result of the binary algorithm on two different levels of zoom-ins for the anonymised image in Figure 9a(iii). More precisely, in (**a**) we show an approx. 40% zoom-in and in (**b**) an approx 70% zoom-in of the original image in Figure 9a(iii).

*Second, the trained data were relatively small and might not contain all possible types of lesions and anatomical regions where such lesions can occur (e.g., the eye or close to the eye; see Figure 1). Note that these aspects can impact the performance of the model, as exemplified in Figure 10, where the algorithm classified the lesion as “malignant“ (see Figure 10a) when the lower part of the iris and the sclera is obvious in the zoom-in while classifying the lesion as “benign” (see Figure 10b) when only the lower part of the sclera is obvious in the zoom-in.*

### 3.4. Keloid vs. Similar-Looking Malignant Lesions

As mentioned in the Introduction, previous studies have shown that malignant lesions such as basal cell carcinoma and squamous cell carcinoma are sometimes misdiagnosed as keloid [72,73,74]. In this section, we trained the algorithms to be able to differentiate between these skin lesions, and hence, we restricted ourselves only to the basal cell carcinoma and squamous cell carcinoma images in [34], as well as to the keloid dataset from [44] for training, validation, and testing. We returned to all 10 models and trained them on augmented data as well as fine-tuned them (since this approach produced the best performance results). The results are presented in Table 21. We see here that DenseNet121 outperformed the rest of the models except in terms of the AUC score, where it is the fourth-best model. The best AUC score was given by the VGG16 model, followed by InceptionNetRNV2 and EfficientNetV2L. Moreover, the results in this table show that VGG16 is in top three algorithms that can differentiate between keloids and other similar-looking skin lesions.

**Figure 11:**
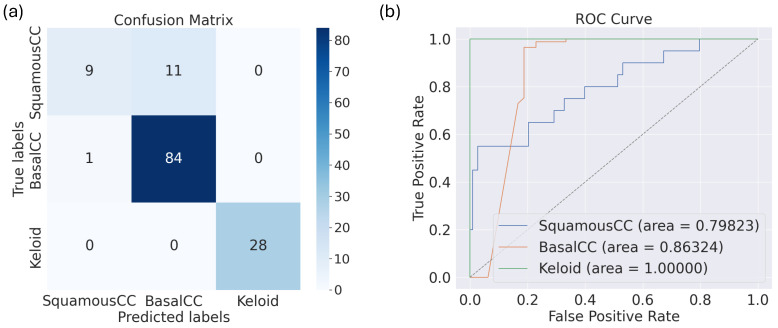
The (**a**) confusion matrix and (**b**) AUC-ROC curve for VGG16 trained on the augmented train data, validated on the original validation dataset (while fine-tuning the base model), and tested on the test data.

Finally, in Figure 11 we show the confusion matrix of the performance of DensNet121, as well as its AUC-ROC curve and score.

## 4. Discussion and Research Limitation

### 4.1. Discussion

In this study, we trained nine classical base CNN models (VGG16, MobileNet, DenseNet121, InceptionV3, EfficientNetB0, Xception, InceptionRNV2, EfficientNetV2-L, and NASNet-L) to perform three classification tasks using two publicly available image datasets. The first classification task was to train the models to classify keloids, melanomas, basal cell carcinomas, squamous cell carcinomas, seborrheic keratosis, nevus, and actinic keratosis as either “malignant“ or “benign” skin lesions. The second task was to train the models to classify these skin disorders as “malignant“, “benign”, or “keloid“, since we wished to distinguish the keloids from other benign lesions. The third classification task was to distinguish keloids from other malignant look-alikes, which sometimes have been misdiagnosed. For the three classification tasks, we employed transfer learning techniques (see Figure 3) to compensate for insufficient image data in biomedicine. It is important to emphasise that the images considered in this study were non-dermatoscopic clinical images (as given by the PAD dataset [34] for benign and malignant lesions and by the Kaggle dataset [44] for keloid lesions), as we aimed to train these models such that they can be used in communities where dermatoscopes are either rare or unavailable and where patients might not be able to have appointments with specialist dermatologists. Note that most of the studies on benign-vs.-malignant classification of skin lesions use the more popular and much larger ISIC dataset of dermatoscopic images from [75,76] or HAM10000 data [77].

We trained the models on the original train data, an oversampled training data, and augmented training data, and we validated and tested them on the original validation dataset and the test dataset (with this one never being exposed to the models during training). For model performance, we used the following classical metrics: accuracy, precision, recall, ${F1}_{score}$, AUC-ROC, and confusion matrix.

### 4.2. Research Limitations

As much as our proposed model performed well on the test data, we admit it had some limitations when tested on some anonymised clinical images obtained from Dr. Brice Chatelain, as shown in Figure 10. Some of the limitations of this study, especially those of the proposed model, have been highlighted in Remark 1. In what follows, we highlight our perceived limitations of this study, including those already highlighted in Remark 1:

- The image dataset used in this study contained non-dermatoscopic (clinical) images of keloids, some malignant skin cancers/lesions, and other benign lesions (including melanomas, basal cell carcinomas, squamous cell carcinomas, seborrheic keratosis, nevus, and actinic keratosis); hence, it may not perform well when tested on other skin lesions not present in training data or dermatoscopic images of skin lesions present in training data.
- In addition, as we mentioned in Section 2.1, not all data we used were pathologically validated (especially the non-cancerous lesions), which could have impacted the classification results we obtained.
- In Remark 1, we highlighted that the models performed poorly on images taken at a long distance from the skin lesion, as the models were trained on images focused on the skin lesions. Also, zoomed-in images of the same original pictures led to blurred images and possible misclassification.
- Lastly, as previously mentioned in Remark 1, the trained data were relatively small in number and might not contain all possible anatomical regions where such lesions can occur (e.g., the eye or close to the eye; see Figure 1). Note that these aspects can impact the performance of the model, as exemplified in Figure 10, where the algorithm classified the lesion as “malignant” (see Figure 10a) when the lower part of the iris and the sclera is obvious in the zoom-in, while classifying the lesion as “benign“ (see Figure 10b) when only the lower part of the sclera is obvious in the zoom-in.

## 5. Conclusions

The results showed that in first two classification tasks, VGG16 outperformed the rest of the models after fine-tuning (see Table 13 and Table 20), while for the third classification task, DenseNet121 outperformed the rest of the models. The VGG16 model (i.e., the best models for the first two classification tasks) was also tested on three new clinical images (graciously provided by B. Chatelain), which were not available on the public datasets used for training/validation/testing. We showed that VGG16 performed well on the binary classification of the original images but not on the classification of the zoom-in images (where at least one image was misclassified). This is probably the result of a lack of similar data (from similar anatomical regions) used for training. Hence, more image data available for training will likely improve the results. Moreover, combining the clinical features of each skin lesion with the images of these lesions has been reported to improve the performance of CNN models [27]. We will consider such an approach in the future (knowing that at this moment only the benign/malignant dataset [34] also contains clinical features; the keloid dataset [44] does not contain such detailed information). As mentioned in the Introduction, we also plan to further explore the use of vision transformers (ViTs), as they show potential in image classification, since they use fewer computational resources compared to CNN models, even though they do not currently outperform the CNN models [39], especially when trained on small datasets. We also hope to delve into mechanistic learning approaches, where we can leverage knowledge-driven modelling with data-driven modelling to improve the interpretability of such models [78]. We will also explore the ablation study of each considered architecture and further investigate how feature selection/extraction using algorithms such as principal component analysis (PCA), minimum redundancy maximum relevance (mRMR), autoencoder (AE), linear discriminant analysis (LDA), etc., could improve model performance and computational efficiency. We will also explore how the use of Generative Adversarial Network (GAN) for data augmentation [28] affects the performance of the models.

## 6. Comparison with Published Literature

As we mentioned in the Introduction, we are not aware of any studies classifying keloid images among benign and malignant skin lesion images. However, some studies classify other skin lesions using dermatoscopic data [79,80] or non-dermatoscopic data [27,81]. To position our study within the published literature, we compared our results with those in studies that equally classify the skin lesions as either malignant or benign. In [79], a CNN-based model was trained on the publicly available dermoscopic datasets of skin lesions [77,82,83] to classify benign vs malignant skin lesions and returned an accuracy of 0.854±0.032, a precision of 0.936±0.017, and a recall of 0.88±0.048. The same study [79] also considered a machine learning-based approach and arrived at an accuracy of 0.738±0.011, a precision of 0.854±0.01, and a recall of 0.811±0.013. In [80], a deep learning (DL) model was built using a public dermatoscopic dataset from [77] and a Kaggle dataset from the ISIC archive [35], and the researchers obtained an accuracy of 0.88, a precision of 0.93, a recall of 0.83, and an F1 score of 0.88. Our proposed model for the binary classification based on the VGG16 model (see first row in Table 17) yielded an accuracy of 0.85, a precision of 0.86, and a recall of 0.77. It is important to emphasise that the models in [79,80] were trained on dermatoscopic image datasets (which are more available publicly, thanks to the ISIC and HAM10000 datasets, which, when combined, have over 400,000 images of different skin lesions as of 2024). In this study, our focus was to train a deep learning model on non-dermatoscopic images of skin lesions to provide ways of diagnosing these skin lesions using just phone camera pictures (clinical images) in areas where dermatoscopes are not available or are too expensive to consider. To the best of our knowledge, very few machine learning and deep learning models have been used to classify skin lesions using non-dermatoscopic images [27,81], with [34] (i.e., the dataset used in [27]) being the only one available publicly. While [27] considered similar datasets, we emphasise that we added a keloid dataset from Kaggle [44]. In [27], the clinical images and the clinical features provided in the metadata were combined, while in this study, we only considered the clinical images, and the addition of the clinical features will be a focus of future work. The performance of the multiclass classification model in [27], when clinical images were combined with clinical features (i.e., patient information), yielded an accuracy of 0.788±0.025, precision of 0.800±0.028, a recall of 0.788±0.025, and an F1 of 0.790±0.027; our proposed model for the binary classification (i.e., benign vs. malignant) yielded an accuracy of 0.851, a precision of 0.860, a recall of 0.775, and an F1 of 0.815; the multiclass VGG-based model (i.e., benign vs. malignant vs. keloid; see Table 20), yielded an accuracy of 0.877, a precision of 0.881, a recall of 0.877, and an F1 of 0.878; and finally, the multiclass DenseNet-based model (i.e., keloid vs. basal cell carcinoma vs. squamous cell carcinoma; see Table 21), yielded an accuracy of 0.910, a precision of 0.911, a recall of 0.910, and an F1 of 0.897. In Table 22, we present a summary of the comparisons with previous work.

It is evident that for a model based on less than 3000 non-dermatoscopic images, our model has better performance, with an ≈9% increase in performance compared to [27], as seen in Table 22. We recognise that our models were trained for fewer classes (i.e., two and three classes) compared to the six-class models in [27], which might explain the difference in the results.

## Figures and Tables

**Table 1:** Summary of some previous studies on medical image classification.

Study	Dataset	Algorithm	Category	Results	Limitation
[28]	Liver lesion images (CT scans)	GAN + CNN	Benign vs. Malignant Liver Lesions	Improved CNN accuracy with GAN augmentation (AUC-ROC = 0.93, recall = 0.857, specificity = 0.924)	GAN-generated images may not fully capture real variations; limited dataset size
[27]	Non-dermatoscopic skin lesion images + clinical data	Several CNN classifiers	Skin Cancer Detection	Accuracy = 0.788 ± 0.025, precision = 0.8 ± 0.028, recall = 0.788 ± 0.025	Dependency on patient metadata; limited generalisation ability
[29]	Mammogram images tested on the MIAS and DDSM	ResNet101 + Metaheuristic optimisation	Breast Cancer Classification	DDSM (Accuracy = 0.986, recall 0.987) MIAS (accuracy = 0.992, recall = 0.979)	High computational cost; possible overfitting
[31]	HAM10000 + ISIC dataset	proposed CNN-based SkinNet-16	Benign vs. Malignant Skin Lesions	Accuracy ≈ 0.992, recall ≈ 0.98	Limited dataset; only binary classification
[32]	Post-operative scar images + clinical data	Proposed CBAM + ResNet50	Scar Severity Prediction	Accuracy = 0.733, AUC-ROC = 0.912, recall = 0.733	Variability in scar assessment; need for real-world validation
[33]	Keloid images (Laser Speckle Contrast Imaging)	Proposed a cascaded vision transformer architecture	Keloid Severity Evaluation	Average accuracy = 0.927	Limited dataset; requires clinical validation

**Table 2:** Pathological classification of the skin disorders image dataset in [34], detailing the total number of images available for each skin disorder, the total images classified as malignant or benign, and the overall number of images in the dataset.

Diagnostic Class	Skin Disease	Nbr. of Images	Total Nbr. Images
	Melanoma	52	
Malignant	Basal Cell Carcinoma	845	1089
	Squamous Cell Carcinoma	192	
	Seborrheic Keratosis	235	
	Nevus	244	1209
Benign	Actinic Keratosis	730	
Total Images			2298

**Table 3:** Classical VGG16 architecture.

Layers	Output Size	VGG16
Convolution	224×224	7×7 conv
Pooling	112×112	2×2 max pool
Convolution	56×56	3×3 conv
Pooling	28×28	2×2 max pool
Convolution	14×14	3×3 conv
Pooling	7×7	2×2 max pool
Fully Connected	4096	Fully connected layer
Fully Connected	4096	Fully connected layer
Output	1000 (classes)	Output layer with softmax activation

**Table 4:** Classical DenseNet121 architecture.

Layers	Output Size	DenseNet121
Convolution	112×112	7×7 conv
Pooling	56×56	3×3 max pool
Dense Block	56×56	6 layers
Transition Layer	28×28	1×1 conv, 2×2 avg pool
Dense Block	28×28	12 layers
Transition Layer	14×14	1×1 conv, 2×2 avg pool
Dense Block	14×14	24 layers
Transition Layer	7×7	1×1 conv, 2×2 avg pool
Dense Block	7×7	16 layers
Pooling	1×1	7×7 avg pool
Fully Connected	1024	Fully connected layer
Output	1000 (classes)	Output layer with softmax activation

**Table 5:** Classical InceptionV3 architecture.

Layers	Output Size	InceptionV3
Convolution	149×149	3×3 conv
Convolution	147×147	3×3 conv
Convolution	147×147	3×3 conv
Pooling	73×73	3×3 max pool
Convolution	71×71	3×3 conv
uses the Convolution	35×35	3×3 conv
Inception Module	35×35	Inception A
Inception Module	17×17	Inception B
Inception Module	8×8	Inception C
Pooling	1×1	8×8 avg pool
Fully Connected	2048	Fully connected layer
Output	1000 (classes)	Output layer with softmax activation

**Table 6:** MobileNet architecture.

Layers	Output Size	MobileNet
Convolution	112×112	3×3 conv
Depthwise Separable Conv	112×112	3×3 depthwise, 1×1 pointwise
Pooling	56×56	2×2 max pool
Depthwise Separable Conv	56×56	3×3 depthwise, 1×1 pointwise
Depthwise Separable Conv	28×28	3×3 depthwise, 1×1 pointwise
Pooling	14×14	2×2 max pool
Depthwise Separable Conv	14×14	3×3 depthwise, 1×1 pointwise
Depthwise Separable Conv	7×7	3×3 depthwise, 1×1 pointwise
Pooling	1×1	7×7 avg pool
Fully Connected	1024	Fully connected layer
Output	1000 (classes)	Output layer with softmax activation

**Table 7:** EfficientNet-B0 architecture.

Layers	Output Size	EfficientNet-B0
Convolution	112×112	3×3 conv, 32 filters, stride 2
MBConv1	112×112	3×3 depthwise, 16 filters
MBConv6	56×56	3×3 depthwise, 24 filters, stride 2
MBConv6	28×28	5×5 depthwise, 40 filters, stride 2
MBConv6	14×14	3×3 depthwise, 80 filters, stride 2
MBConv6	14×14	5×5 depthwise, 112 filters
MBConv6	7×7	5×5 depthwise, 192 filters, stride 2
MBConv6	7×7	3×3 depthwise, 320 filters
Convolution	7×7	1×1 conv, 1280 filters
Pooling	1×1	Global average pooling
Fully Connected	1280	Fully connected layer
Output	1000 (classes)	Output layer with softmax activation

**Table 8:** Xception architecture.

Layers	Output Size	Xception
Convolution	149×149	3×3 conv, 32 filters, stride 2
Convolution	147×147	3×3 conv, 64 filters
Entry Flow	73×73	3 × SeparableConv layers, 128 filters, stride 2
Entry Flow	37×37	3 × SeparableConv layers, 256 filters, stride 2
Entry Flow	19×19	3 × SeparableConv layers, 728 filters, stride 2
Middle Flow	19×19	8 × (3 × SeparableConv layers, 728 filters)
Exit Flow	10×10	3 × SeparableConv layers, 1024 filters, stride 2
Exit Flow	10×10	3 × SeparableConv layers, 2048 filters
Pooling	1×1	Global average pooling
Fully Connected	2048	Fully connected layer
Output	1000 (classes)	Output layer with softmax activation

**Table 9:** InceptionResNetV2 architecture.

Layers	Output Size	InceptionResNetV2
Convolution	149×149	3×3 conv, 32 filters, stride 2
Convolution	147×147	3×3 conv, 32 filters
Convolution	73×73	3×3 conv, 64 filters, stride 2
Inception-ResNet-A	35×35	5 × Inception-ResNet-A modules
Reduction-A	17×17	Transition layer (pooling, conv)
Inception-ResNet-B	17×17	10 × Inception-ResNet-B modules
Reduction-B	8×8	Transition layer (pooling, conv)
Inception-ResNet-C	8×8	5 × Inception-ResNet-C modules
Convolution	8×8	1×1 conv, 1536 filters
Pooling	1×1	Global average pooling
Fully Connected	1536	Fully connected layer
Output	1000 (classes)	Output layer with softmax activation

**Table 10:** EfficientNetV2-L architecture.

Layers	Output Size	EfficientNetV2-L
Convolution	224×224	3×3 conv, 32 filters, stride 2
MBConv1	112×112	3×3 depthwise, 32 filters
MBConv4	56×56	3×3 depthwise, 64 filters, stride 2
Fused-MBConv4	28×28	3×3 fused conv, 128 filters, stride 2
Fused-MBConv6	14×14	3×3 fused conv, 256 filters, stride 2
MBConv6	7×7	3×3 depthwise, 512 filters, stride 2
MBConv6	7×7	3×3 depthwise, 1280 filters
Convolution	7×7	1×1 conv, 1280 filters
Pooling	1×1	Global average pooling
Fully Connected	1280	Fully connected layer
Output	1000 (classes)	Output layer with softmax activation

**Table 11:** NASNet-L architecture.

Layers	Output Size	NASNetLarge
Convolution	224×224	3×3 conv, 96 filters, stride 2
Normal Cell	112×112	5 × Normal cells, 168 filters
Reduction Cell	56×56	Transition layer (pooling, conv)
Normal Cell	56×56	5 × Normal cells, 336 filters
Reduction Cell	28×28	Transition layer (pooling, conv)
Normal Cell	28×28	5 × Normal cells, 672 filters
Reduction Cell	14×14	Transition layer (pooling, conv)
Normal Cell	14×14	5 × Normal cells, 1344 filters
Reduction Cell	7×7	Transition layer (pooling, conv)
Normal Cell	7×7	5 × Normal cells, 4032 filters
Pooling	1×1	Global average pooling
Fully Connected	4032	Fully connected layer
Output	1000 (classes)	Output layer with softmax activation

**Table 12:** Accuracy, precision, recall and F1 score for the pretrained models presented above (rows 1–9). Here, the models were trained and validated on all raw skin lesion data (before any oversampling or data augmentation; see Section 2.1), and before any fine-tuning of the pretrained models. In the “Accuracy” column, we show in bold the largest value indicating the best model.

Model	Accuracy	Precision	Recall	${F1}_{\mathrm{score}}$	AUC
VGG16	0.7893	0.7642	0.7297	0.7465	0.8809
MobileNet	**0.8008**	0.7287	0.8468	0.7833	0.8776
DenseNet121	0.7969	0.7589	0.7658	0.7623	0.8841
InceptionV3	0.7356	0.6721	0.7387	0.7039	0.8123
EfficientNetB0	0.5747	0.0	0.0	0.0	0.5608
Xception	0.7635	0.7333	0.6937	0.7130	0.8280
InceptionRNV2	0.7165	0.6761	0.6396	0.6574	0.8131
EfficientNetV2L	0.5754	0.0	0.0	0.0	0.5943
NASNetLarge	0.6743	0.625	0.5856	0.6047	0.7370

**Table 13:** Accuracy, precision, recall, F1 score, and AUC of the pretrained CNN models (rows 1–9). Here, the models were trained and validated on all raw skin lesion data (before any oversampling or data augmentation; see Section 2.1), after fine-tuning the pretrained base models. In the “Accuracy” column, we show in bold the largest value indicating the best model.

Model	Accuracy	Precision	Recall	${F1}_{\mathrm{score}}$	AUC
VGG16	**0.8467**	0.8142	0.8288	0.8214	0.9274
MobileNet	0.7816	0.7455	0.7387	0.7421	0.8633
DenseNet121	0.8238	0.8495	0.7117	0.7745	0.9039
InceptionV3	0.8122	0.7719	0.7930	0.7822	0.8880
EfficientNetB0	0.8008	0.7706	0.7568	0.7636	0.8793
Xception	0.8161	0.8316	0.7117	0.7670	0.8958
InceptionRNV2	0.8161	0.7838	0.7838	0.7838	0.8793
EfficientNetV2L	0.8314	0.7815	0.8378	0.8097	0.8869
NASNetLarge	0.7203	0.6173	0.9009	0.7326	0.8501

**Table 14:** Accuracy, precision, recall, F1 score, and AUC of the pretrained CNN models (rows 1–9) trained on oversampled train dataset (to address the issue of imbalanced data classes; see Table 1) on the test data. Here, the pretrained base models were not fine-tuned. In the “Accuracy” column, we show in bold the largest value indicating the best model.

Model	Accuracy	Precision	Recall	${F_{1}}_{\mathrm{score}}$	AUC
VGG16	**0.7969**	0.7900	0.7117	0.7488	0.8787
MobileNet	0.7893	0.7154	0.8378	0.7718	0.8674
DenseNet121	0.7893	0.7188	0.8288	0.7699	0.8742
InceptionV3	0.7203	0.6301	0.8288	0.7160	0.8001
EfficientNetB0	0.5785	0.5785	0.2162	0.3038	0.5615
Xception	0.7548	0.7156	0.7027	0.7091	0.8031
InceptionRNV2	0.7471	0.7064	0.6937	0.7000	0.8250
EfficientNetV2L	0.5402	0.4764	0.8198	0.6026	0.6011
NASNetLarge	0.6935	0.6449	0.6216	0.6330	0.7815

**Table 15:** Accuracy, precision, recall, ${F1}_{score}$, and AUC of the transfer learning models (rows 1–9) on the test dataset. Here, the models were trained on oversampled train dataset, and the pretrained base models were fine-tuned. In the “Accuracy” column, we show in bold the largest value indicating the best model.

Model	Accuracy	Precision	Recall	${F1}_{\mathrm{score}}$	AUC
VGG16	0.8237	0.7982	0.7839	0.7909	0.9255
MobileNet	0.7931	0.7822	0.7117	0.7453	0.8668
DenseNet121	0.8084	0.8020	0.7297	0.7642	0.8917
InceptionV3	0.7854	0.7391	0.7658	0.7522	0.8641
EfficientNetB0	**0.8391**	0.8224	0.7928	0.8073	0.8978
Xception	0.8352	0.8269	0.7748	0.8000	0.9031
InceptionRNV2	0.8276	0.8000	0.7928	0.7964	0.8889
EfficientNetV2L	0.8199	0.7963	0.7748	0.7854	0.8591
NASNetLarge	0.7050	0.5966	0.9459	0.7317	0.8762

**Table 16:** Accuracy, precision, recall, F1 score, and AUC of the transfer learning models (rows 1–9) on the test dataset. Here, we trained all models on augmented train datasets (but before fine-tuning of the base models). In the “Accuracy” column, we show in bold the largest value indicating the best model.

Model	Accuracy	Precision	Recall	${F1}_{\mathrm{score}}$	AUC
VGG16	0.8084	0.8081	0.7207	0.7619	0.8808
MobileNet	**0.8276**	0.8367	0.7387	0.7847	0.8965
DenseNet121	0.8084	0.7798	0.7658	0.7727	0.8962
InceptionV3	0.7165	0.6529	0.7117	0.6810	0.7974
EfficientNetB0	0.5057	0.4505	0.7387	0.5597	0.5575
Xception	0.7356	0.6875	0.6937	0.6906	0.8186
InceptionRNV2	0.7356	0.7100	0.6396	0.6730	0.8191
EfficientNetV2L	0.5556	0.4868	0.8288	0.6133	0.6845
NASNetLarge	0.7011	0.6774	0.5676	0.6176	0.7694

**Table 17:** Accuracy, precision, recall, F1 score, and AUC for the transfer learning models (rows 1–9) on the test dataset. Here, we trained these models on augmented train dataset and *fine-tuned* the base models. In the “Accuracy” column, we show in bold the largest value indicating the best model.

Model	Accuracy	Precision	Recall	${F1}_{\mathrm{score}}$	AUC
VGG16	**0.8506**	0.8600	0.7748	0.8152	0.9205
MobileNet	0.7854	0.7570	0.7297	0.7431	0.8728
DenseNet121	0.8467	0.8318	0.8018	0.8165	0.9101
InceptionV3	0.8352	0.8469	0.7477	0.7943	0.9168
EfficientNetB0	0.8199	0.7909	0.7838	0.7873	0.9020
Xception	0.8276	0.8113	0.7748	0.7926	0.9052
InceptionRNV2	0.8200	0.8137	0.7477	0.7793	0.8976
EfficientNetV2L	**0.8506**	0.8461	0.7928	0.8186	0.9197
NASNetLarge	0.8391	0.8556	0.7477	0.7981	0.9026

**Table 21:** Accuracy, precision, recall, ${F1}_{score}$, and AUC of the models on the test dataset. The models were trained on the augmented train dataset and validated on the original validation datasets, with the base models fine-tuned. In the “Accuracy” column, we show in bold the largest value indicating the best model.

Model	Accuracy	Precision	Recall	${F1}_{\mathrm{score}}$	AUC
VGG16	0.8647	0.8674	0.8647	0.8378	0.8982
MobileNet	0.8346	0.8045	0.8346	0.8100	0.8727
DenseNet121	**0.9098**	0.9110	0.9098	0.8972	0.8872
InceptionV3	0.8797	0.8956	0.8797	0.8559	0.8848
EfficientNetB0	0.8421	0.8172	0.8421	0.8133	0.8867
Xception	0.8346	0.8045	0.8346	0.8100	0.8836
InceptionRNV2	0.8647	0.8521	0.8647	0.8447	0.8976
EfficientNetV2L	0.8571	0.8343	0.8571	0.8344	0.8942
NASNetLarge	0.8571	0.8551	0.8571	0.8297	0.8819

**Table 22:** Comparing accuracy, precision, and recall of our proposed models for the three classification tasks with result of previous works.

Authors	Dataset	Category	Accuracy	Precision	Recall
Brutti et al. [79]	Dermatoscopic image dataset from [77,82,83]	Benign vs. Malignant	0.854±0.032	0.936±0.017	0.88±0.048
Bechelli and Delhommelle [80]	Dermatoscopic image dataset from [35,77]	Benign vs. Malignant	0.88	0.93	0.83
Pacheco and Krohling [27]	Non-dermatoscopic image dataset from [34]	Multiple (six) skin lesion class	0.788 ± 0.025	0.8 ± 0.028	0.79 ± 0.027
Udrea et al. [81]	Non-dermatoscopic image dataset (private)	Unspecified	unavailable	unavailable	0.951
		**Benign vs. Malignant;**	**0.851**	**0.860**	**0.775**
**Ours**	**Non-dermatoscopic image dataset from** [34] **and** [44]	**Benign vs. Malignant vs. Keloid;**	**0.877**	**0.881**	**0.877**
		**Keloid vs. BCC vs. SCC**	**0.91**	**0.911**	**0.91**

## Data Availability

The code for this project is available at https://github.com/OEAdebayo/skin-project.

## Appendix A. Details of the Performance Metrics

As explained in Section 2.2.3, we made use of the following performance metrics:

- Accuracy, which measures the proportion of correct predictions made by the model defined as
  (A1) $$\mathrm{accuracy}=\frac{\mathrm{TP}+\mathrm{TN}}{\mathrm{TP}+\mathrm{FP}+\mathrm{TN}+\mathrm{FN}}.$$
- Precision, which measures the ratio of TP predictions among all positive predictions (TP and FP), is given by
  (A2) $$\mathrm{precision}=\frac{\mathrm{TP}}{\mathrm{TP}+\mathrm{FP}}.$$
  This metric indicates how many of the predicted positive cases are correctly identified.
- Recall (also known as sensitivity or TP rate), which measures the proportion of correctly predicted positive cases (TP) out of all actual positive cases (TP + FN), is given by
  (A3) $$\mathrm{recall}=\frac{\mathrm{TP}}{\mathrm{TP}+\mathrm{FN}}.$$
  In other words, it denotes how many of the actual positive cases were correctly identified by the model. Note that using precision and recall separately might not provide the full picture, since these metrics are subjective of the classification task at hand. Thus, it is better to use metrics that combine both precision and recall.
- A metric that combines the precision and recall is the ${F1}_{score}$. It provides a good evaluation of model performance and is defined below as
  (A4) $${F1}_{score}=2\frac{\mathrm{precision}\times \mathrm{recall}}{\mathrm{precision}+\mathrm{recall}}.$$
  It will not account for imbalance in the dataset.
- We can also evaluate the performance of each model using the Area Under the Receiver Operating Characteristic Curve (AUC-ROC). The AUC-ROC measures the ability of a classifier to differentiate between the positive and negative classes across different threshold values, while the ROC curve itself is a plot of the FP rate (*x* axis) against the TP rate (i.e., the recall on the *y* axis).
- The confusion matrix is another (visual) metric that describes the performance of a classification model by comparing the actual values with the predicted values.

## Appendix B. Hardware and Software Specification

We implemented our models on a high-performance computing (HPC) cluster with compute nodes equipped with dual Intel Xeon Silver 4314 CPUs, where each CPU has 16 cores (totaling 32 cores per node) with varying node RAM capacity and operates at a base frequency of 2.4 GHz, with a maximum turbo frequency of 3.4 GHz. The software stack included Ubuntu 20.04 LTS OS, Python 3.12, and Keras 3.3 with a TensorFlow 2.16.1 backend. The training jobs were submitted via Slurm 19.05.5, and the jobs were assigned based on node availability. The jobs were run without GPU acceleration, relying entirely on CPU-based training. The rest of the software dependencies can be found in the requirements.txt file available in the GitHub link provided in the “Data Availability Statement” above.
